# Faculty development strategies to empower university teachers by their educational role: A qualitative study on the faculty members and students’ experiences at Iranian universities of medical sciences

**DOI:** 10.1186/s12909-023-04209-0

**Published:** 2023-04-19

**Authors:** Sima Ghasemi, Leila Bazrafkan, Arash Shojaei, Tayebeh Rakhshani, Nasrin Shokrpour

**Affiliations:** 1 Shiraz University of Medical Sciences, Shiraz, Iran; 2 Clinical Education Research Center, Shiraz University of Medical Sciences, Shiraz, Iran; 3 School of Medicine, Shiraz University of Medical Sciences, Shiraz, Iran; 4 Clinical Education Research Center, Shiraz University of Medical Sciences, Shiraz, Iran

**Keywords:** Teacher development, Teacher professional, Strategies faculty development, Learning styles, Task

## Abstract

**Background:**

This study aimed to identify and explain the strategies of faculty development based on their role at Iranian Universities of Medical Sciences.

**Methods:**

We conducted a qualitative content analysis study in 2021 using purposive, snowball sampling, with a maximum variation in the faculty members’ age and experience level. Twenty four participants were enrolled in this study (eighteen faculty members and six medical sciences students); data collection consisted of two phases of semi-structured interviews and a brainstorming group technique. Data were categorized into two themes and six related subthemes, based on their similarities and differences after frequent summarization.

**Results:**

The data analysis yielded two themes and eight categories. The first theme was to explain competencies based on role and task with two sub-themes: Tasks and capabilities and development and excellence of personal qualities. The second theme was the best strategies for empowering the teachers with four sub-themes, including problem-based learning, integration of methods, evaluation-based education, and scholarship in education (PIES), which explains the strategies that can support the development of teachers in medical sciences universities, and all the concepts were interrelated to each other.

**Conclusion:**

From the experiences of faculty members, the importance of some strategies in education and empowering the teachers’ professional competence dimensions should be emphasized. PIES could explain the practical strategies that can support the development of teachers in medical sciences universities.

**Supplementary Information:**

The online version contains supplementary material available at 10.1186/s12909-023-04209-0.

## Introduction

The quality of education in universities depends on the faculty members’ competence and dynamism. This is in direct relationship with the faculty members’ empowerment since it leads to the promotion of their competence, hence leading to the promotion of universities and students [1–3]. Moreover, education experts believe that empowerment programs for the faculty members should be designed in a way that it provides them with the necessary abilities for their main role in education [4, 5]. Steinert (2016) states that the efficiency and empowerment of teachers are the basis for the efficacy of educational outcomes. Empowerment of the faculty members might be an approach to changing the teachers’ efficacy, which indirectly affects the students’ learning [6].

Empowerment is a multidimensional social process through which people can regulate their lives and get empowered by attention to the issues important to them. Empowerment of employees is a set of systems, methods, and activities utilized through development of individuals’ capabilities and competence aiming to improve and increase the productivity, growth, and development of the organization and human resources based on the organizational goals [7–9]. Various studies have stated that the teachers’ performance is related to their competence and capabilities [10–12].

However, few empowerment programs are directly related to their duties and activities. Weaknesses in methodology prevent these programs from reaching definite outcomes, while empowerment programs for teachers can have a great role in the promotion of universities [6, 9, 13]. Harden and other researchers emphasize that a teacher or lecturer has different roles and tasks, for which he/she must be prepared to play an influential role, and a teacher training mechanism is needed [12, 14, 15]. Faculty development refers to the activities designed to help medical sciences teachers improve their competence in teaching and learning in areas such as knowledge, skills, and attitude which are vital for their different tasks and roles[12]. University teachers have many duties and roles, which are interrelated, and their inability to perform any of these tasks or roles affects their total activities as faculty members[4, 12]. One of the most important roles the faculty members have is education, in which they have 6 roles and 11 duties. To empower the academic staff, the authorities should take measures to strengthen these roles. Wilkerson and Irby state that a comprehensive faculty development platform should consist of [1] professional development, [2] instructional development, [3] leadership development, and [4] organizational development which empowers the faculty members to be best in their roles as edcators [9].

There are several methods used for empowering the university teachers, each being based on a specific criterion [14, 16, 17]. All institutions and individual disciplines attempt to increase the medical teachers’ and administrators’ skills in the performance of their current responsibilities and continuously enhance and redefine their competencies while acknowledging that individual faculty members cannot excel at all of the recognized responsibilities [18, 19].

These strategies have initiated the theoretical foundations in learning practices, resulting in improved self-efficacy and commitment in various educational scholarship activities [20]. The attainment of a medical teacher qualification has been associated with development of the roles, leadership positions, and expanded responsibilities in medical education [21]. The medical teacher also reports a need to transfer his/her academic learning into the educational practices in the workplace, which may lead to particular challenges and tensions that are still under-explored [20].

The previous research conducted in the last decade on the academic staff reveal that professional development of the academic staff is not promoted sufficiently; therefore, it is vital to clarify the faculty members’ needs and plan to meet them [22]. Given that the academic staff’s empowerment and recognition of their needs is affected by the educational functioning and the culture of the universities, it seems necessary to conduct studies aiming at surveying the academic staff’s needs as to their educational responsibilities and using appropriate strategies to meet their needs. A survey of empowerment programs indicated a clear mindfulness in each program, so that various strategies were employed to satisfy the needs and and attain the educational goals [23].

Empowerment will not be effective when not considering the strategies in teaching and learinig; the need for using these important strategies should be sought in the faculty members’ experiences, so this need a qualitative study. No study was found on strategies used for the faculty members’ empowerment based on their roles and duties; therefore, the results of the present study can help all those involved in teahing in universities.

This study aimed to explain the educational empowerment strategies of teachers based on the tasks, and roles of medical teachers at Iranian Universities of Medical Sciences.

## Methods

### Design

This study aimed to explain the strategies of faculty development based on their roles and duties at some Universities of Medical Sciences in Iran. This qualitative study used content analysis and brainstorming techniques (BST). Data collection consisted of two phases of semi-structured interviews and brainstorming group techniques (24–25). We used the empowerment model proosed by thorndike et al. (2006) which describes the design, implementation, and evaluation of the Junior Faculty Development Program. This model benefits the institutions to empower their faculty [26].

### Setting and sampling

The history of Shiraz University of Medical Sciences (SUMS) School of Medicine dates back to December 1946 when an “Institute of Health for Higher Education”, which was affiliated to Tehran University, was established in Shiraz. Medical Educational Development Center (EDC) of SUMS was established in 1972 as a center affiliated to the vice-chancelery of educational affairs. EDC was established as a center for planning, policy-making, development, and promotion of educational activities such as curriculum planning, evaluation, and research. This research was conducted in Shiraz University of Medical Sciences, a well-known teacher training center in Eastern Mediterranean Regional Office (EMRO), [27]. The participants consisted of 18 faculty members and six medical sciences students. They were selected using purposeful sampling while considering maximum variations in age, discipline, and experience in different universities of medical sciences in Iran (Shiraz, Fasa, Jahrom, Iran, Tehran, Mashhad, Ahvaz, Kermanshah, and Tabriz). To complete the data, we selected some students to take part in the study. In qualitative studies, the data obtained determine who we should be involved in the next parts of the study, so that the researcher can reach data saturation. In this study, we asked the faculty members about the temporary strategies of teaching and learning, one of them stated that teaching is a mutual activity and the leaners’ views should be taken into consideration. That’s why we selected six students to be interviewed as well. Sampling and data coding continued till data saturation when no new code was obtained during the interviews a nd repetition of the previous categories and codes. The participants who participated in the study were in a spectrum of weak to strong as to the mean scores, extracurricular activities, and research.

The inclusion criteria for the faculty members were their rich experience and familiarity in medical teacher tesk, educational stertegy in empowering, a minimum of 8-10-year teaching experience, and their willingness to participate in the study. The exclusion criterion was the faculty members’ unwillingness to participate in the study. Moreover, the inclusion criteria for medical students were studying in the secound year of medical sciences discipline and willingness to participate in the study. Initially, we collected data from a faculty member well known for his high-quality teaching; then, we continued data gathering from other faculty members and medical students until data saturation was achieved [37–40].

### Data collection

#### Phase one

In the first step, we invited the participants (eighteen faculty members and six medical sciences students) for semi-structured interviews from all Universities of Medical Sciences in Iran to take part in the interview, each lasting from 20 to 65 min approximately; based on the conditions and participants’ responses, we performed the interviews in person or through chat on social networks like WhatsApp.

In this stage of the study, we first asked the academic staff about their roles, task, preferred teaching method, and the strategies to empower teacher. We asked them to describe the way they established a relationship with their students, so that they could could promote their learning abilities. Here, we needed to ask them what their sterategy in learning and teaching methodology was based on their reports about the students. The interview guide is displayed in the Appendix.

In the second phase of this research, focus group discussions and brainstorming group techniques (BST) were applied to gather data on the participants’ experiences about strategies to empower the teachers and improve the faculty development in Shiraz with 14 faculty members, who accepted to take part in this part of the study as well [26]. All the focus groups were directed by the main investigator, each lasting 2.5 h, with 10 participants in every session. The participants were selected purposefully from those who were already interviewed; they sat around a circle desk to express the priorities as to the strategies of faculty development in universities of medical sciences. In the beginning, the written questions were presented to every member, and they reflected their viewpoints to the group.

The group’s opinions were discussed to clarify the concept and make it more understandable for the assessment process by mind mapping. In this part of brainstorming, we attempted to catch every idea and circulate it through the members. In this creative manner, we used a non-linear diagram to ensure the inflow of ideas, so we did not miss any idea. We started this section with a question in the center of the mind map, “What are the strategies used for improving faculty development?“, to include every participant’s thoughts. As to the results of the first stage, one of the reseachers explained in detail that initially most of the staff preferred the convergent teaching style. This style consists of two abstract conceptualization and active experimentation learning style. Those with convergent style are interested in logical reasoning and inductive argument. These individuals are more successful in problem solving, decision making, and practical implication. They gain knowledge using a specific method through hypothetical-deductive reasoning, which focuses on solving a specific problem.

Then, they discussed about Harden’s article entitled “The good teacher is more than a lecturer: The twelve roles of a teacher”. The participants responded the question and presented their views about preferable strategies for empowerment of the faculty members considering the dominant convergent learning style and the faculty members’ roles, responsibilities, meta-competence, and their ethical and professional features.

In addition to the coordinator, who was selected from one of the research team members, a colleague was asked to carefully write down the participants’ ideas and their verbal and non-verbal reactions. The recorded discussion of every session was transcribed verbatim immediately after recording and then analyzed. The texts obtained from the interviews and focus groups were monitored repeatedly to perceive the general understanding. Finally, meaning units or primary codes were extracted [28].

### Data analysis

Data were analyzed through the conventional content analysis method. It is a specific sort of scientific data processing used to find the actual words in a text, so that the data can be summarized, described, and interpreted. We iteratively recognized the themes and sub-themes for each interview, and the sub-themes were later compared and contrasted across all the interviews. Therefore, the researchers identified the main key subjects based on the recognition of their development. Then, they provided the framework of the subject. In the next step, all the manuscripts derived from the summary forms of the individual and group interviews were reassessed, rewritten, and arranged according to the obtained subjects’ framework. After comparing the observed associations, concepts, contrasts, and similarities, the two researchers had no conflict with the extracted themes [29].

### Rigor

The rigor or trustworthiness of data was approved using Guba and Linkon’s criteria. The trustworthiness or rigor of the study method and process was ensured using different methods, such as prolonged participation, purposive sampling, peer interpretation, member review, and audit follow-up. We used the researcher involvement in the data using long-term involvement and close contact with participants to achieve validity. We used a purposive sampling of the faculty members and a wide range of participants from different disciplines and experiences to improve the transferability of the results. In addition, to check the methods of data collection and analysis, as well as the study’s compliance with the research protocol, we used the audit direction by two academic experts. We also sent the results and preliminary data to the participants for approval. The method and procedure of the research plan were reviewed and approved by the research ethics committee of the university. All stages of the research including data collection, storage, analysis, and reporting were confidential and the data were only available to the research team members. Confirmability was also attained by observing several interviews, manuscripts, codes and sub-themes and informing the individuals of other researchers’ points of view. The transferability of the study was determined by the precise and purposive explanation of the processes and activities. We also followed the study processes and characteristics of the studied population [30].

## Results

In this study, the mean age of the faculty members was 45.34 ± 12.60 years and all of them were married. From 18 faculty members, 10 (56%) were males and 8 (44%) were females. The work experience of the faculty members ranged from 8 to 25 years of teaching in Iranian medical sciences universities. The participants consisted of 16 (89%) medical, dentistry, nursing, health sciences school faculty members and 2 (11%) experts in medical education and other schools; of the faculty members, 3 (17%) were assistant profesor, 7 (39%) were associate profesor, and 8 (44%) were ful profesor.

Two themes and six interrelated sub-themes emerged from the data. (Table 1)

**Table 1:** Themes and subthemes in empowering the university professors

Themes	Subthemes	Codes
**Explain competencies based on role**	Tasks and capabilities	Mastery of the subject Pre-teaching abilities Knowledge of learning patterns Holding a fair exam The power of expression, comprehension, and transmission of educational materials Determination and seriousness in classroom management Problem-solving skills Critical thinking Clinical reasoning Ability to access new information Audit and self-assessment Entrepreneurial skills Team building and teamwor k
	Excellence in personal qualities	Having a generous chest and good mood Being a role model Maintaining the student’s personality and respecting him Being rational and a master of criticism Humorous natural master Non-discrimination between students Seeking justice Conflict management Advanced communication skills
**Best strategies in empowering teachers**	Problem-based training	Learning high levels of cognition, group learning, peer learning, mentoring flow in teamwork, motivation, self-management
	Integration methods	Combination of simultaneous and asynchronous teaching methods, the combination of subject and content, combination of teaching methods,
	Evaluation-based training	Assessment to motivate, provide feedback, improve learning, emphasize supervision, apply what has been learned
	Scholarship in teaching	Creating a field of science in practice, influencing the educational environment, improving the level of education and educational research in the university, promoting educational science, promoting students’ satisfaction

### Results

The data analysis yielded two themes and eight subthemes. The first theme was to explain competencies based on role and task with two sub-themes: Tasks and capabilities and Development and excellence of personal qualities. The second theme was the best strategies for empowering the teachers with four sub-themes, including problem-based learning, integration of methods, evaluation-based education, and scholarship in education (PIES), which explains the strategies that can support the development of teachers in medical sciences, and all the concepts were interrelated.

### Theme 1: explaining competencies based on roles

Based on the experiences of the study participants in the process of empowering teachers, in addition to learning style, their roles and tasks should be considered. To play their different roles, teachers need competencies and traits, some of which are among their roles as academic staff.

### Tasks and capabilities

Participants defined the roles based on Harden’s study on faculty members’ roles. They expressed the competencies required for the teachers in the academic staff position based on a three-circle model of Dandy experiences. From their experiences and opinions, the teachers and experts considered the features and attributes necessary to play the role of a teacher. Problem-solving skills, critical thinking, and clinical reasoning skills were necessary, and priority was given to the essential problem-solving skills in teacher and student training. One of the teachers said in this regard:

*“…In my experience, a good teacher is a good educational designer, teacher, and a good evaluator. They are all combined to make a good lecturer…”* (Participant No: 12).

### Tasks and capabilities

#### Development and excellence of personal qualities

Based on their own experiences and the statements of other teachers, the participants each addressed the humanistic traits that a university professor should possess. These characteristics include having a good temper, considering the student’s personality and respecting him, and being rational, criticizable and humorous. Practicing no discrimination between students; seeking justice; managing the conflicts; having advanced communication skills; especially being a role model for students and colleagues, which means that teachers with good morals and humanistic qualities; and creating an academic and ethical atmosphere to guide the student in science and ethics were the other characteristics mentioned by them. In this regard, one of the teachers said:

*“…my teacher always did what he said.“ My dear, try to analyze your behavior and see what you did that created such a result. Come on, try to do your tasks on time! But you never act on time….“* (Participant No 8).

### Theme 2: best strategies in empowering teachers

Based on the statements and experiences of the study participants in empowering teachers,, it is necessary to design better strategies in empowering teachers, so that the mentioned competencies can be achieve. However, empowering the teachers to play different roles should be based on a strategy in line with Shiraz University of Medical Sciences’ dominant learning styles in the first phase of the study.

### Problem-based education

Based on the experiences of the teachers and experts, however, problem-based teaching is not a new method. Teachers believe that, in addition to the involvement of the learners and active and profound teaching knowledge, problem-based education leads to an increase in the learners’ intrinsic motivation, peer-assisted learning, and repeated use of what has been taught in the process of learning. One of the teachers said in this regard:

*" … When I remember, I feel good about it; it was something that was both fun and learning. I think it was done for the first time problem-based training, problem-solving tips that I remember;* …” (Participant No: 5).

Based on the teacher’s experience, in small working groups, they get familiar with the culture of teamwork, group behavior, and that of individual members as well. This is an effective practice in understanding the students in the classroom and group, and they experience and learn how to interact with students. In small groups, the training is based on the problem of the young teachers practicing coaching and seeing the role of mentors. One of the teachers said in this regard:

…” *We have tightly entangled ourselves with busy work and other involvements, a routine life without joy and presence of friends and colleagues, while medical sciences as a whole require collective activities and cooperation; in the workshops, we can get together and benefit from friendship and association*…” (Participant No 7).

### Integration of methods

There are several forms of blended learning in the participants’ experiences. The first is a combination of face-to-face and e-learning, which uses information and communication technologies and face-to-face classes for learning of seekers of science. The integrated approach in continuing education has been a successful method that causes satisfaction and improves the performance of its target population. In this regard, one of the teachers maintained:

“…*We need to decide how our teaching needs are met in a class and how students can learn in different ways when attending classes. I always used this method when the students took part in their learning and usually did their best. You see, in this time of the prevalence of Corona, don’t we have the same approach to teaching? As an academic staff member, I know that everything you tell me does not require physical attendance, so what are presence and absence? You use both e-learning and presence …”)* (Participant No: 11).

Another experience is the integrative use of materials, topics, and educational stuff; for example, an academic staff can apply the curriculum, educational evaluation, and management of the material in combination. One of the teachers said:

“…*What is the meaning of being a professor in any field? They are the ones who can respond our questions correctly. Surface replies can be found in any text, but we expect the lecturer to go beyond the text*.” (Participant No 15).

### Intagration of methods

#### Evaluation-based education

The study participants acknowledged that evaluation was an essential part of the learning process. We should evaluate the teacher’s performance in practical programs. One of the teachers said in this regard:

“ When I see the mere presence without even thinking about the program is enough, I do not even bother thinking; I do not do so. However, when I knew I had to answer, I knew someone was auditing. I am so interested in participating in the program. Thinking about the reason why I cannot act like my colleagues prompts me to be active… The question is whether I can have a hands-on approach in my teaching…” (Participant No 6).

The students also emphasized the importance of the professor’s evaluation, that many of their learning problems are caused by incompetent professors, and empowerment programs are necessary. One of the students said:

“The evaluation of the professor should be improved. The professor plays an essential role in the student’s learning process. There should be a difference between the professor and the student’s learning in terms of the evaluation score, so that the incompetent professor tries to improve his ability. (Participant No: 23).

The faculty members have an important role in the students’ learning, so their evaluation should be revised. The assessment score of the faculty members who are indifferent to the students’ learning should be different from those who do not care about this important issue, so that teachers with low abilities in teaching try to get more empowered.

### Intagration of methods

#### Evaluation-based education: Scholarship in education

Although science studies are one of the concepts that are the last activities after entering the university, the matter and specific knowledge and scholarship of teaching would involve serious and active teachers in promoting educational activities. Activity Knowledge Scholarship of Teaching includes all activities in education at different levels, including teaching; designing educational programs and guidance; counseling; practicing leadership; and training. One of the teachers said in this regard:

“…*a significant impact in my experience is involving teachers in educational activities, and I see how people who were previously indifferent are now active and prepare educational interventions and ideas and ask* …) ( Participant No10).

As to the importance of paying attention to empowering and encouraging teachers, one of the medical students said : “…I think most of the professors in medical sciences, or rather our professors, still tend towards traditional authoritarian teaching styles and do not accept student-centeredness. Some professors have adapted themselves to new methods and are interested in active teaching with students. We should give credits to good teachers to encourage them…”(Participant No: 20).

The evaluation of the professor should be improved. The professor plays an essential role in the student’s learning process.

There should be a difference between the professor and the student’s learning in terms of the evaluation score, so that the weak professor tries to improve his ability.

### Critical approach

The educational process must be evaluated critically. Suppose the process is not criticized, so its problems and shortcomings are not seen in the field to be implemented. In that case, it will have problems in implementation, and the basis for this criticism is effective communication with students, colleagues and experts; one of the participants in this field said :

*“… Small universities are also active, but their activities are not highlighted, while larger universities have lots of facilities and a studio* …; *therefore, the potentials for activities in scholarship are not similarly existent.*“ (Participant No: 7).

## Discussion

This study was conducted to explain the empowerment strategies of teachers at Iranian Universities of Medical Sciences, based on their different roles in the faculty. They emphasized the importance of the lecturers’ roles and duties and considered them more important in determining the academic staff’s competence. The result of the second stage was the study of two themes: explaining the competencies and using more efficient strategies in empowering teachers, each of which having subclasses. The theme of role-based competency explanation includes two subcategories of tasks and capabilities and excellence of personal traits. The theme of superior strategies in teacher empowerment includes problem-based education, combination education, evaluation-based education, and promotion. The scholars explained the top strategies in teacher empowerment.

Based on the present study results, the duties and capabilities of university teachers are based on their roles and preferred learning style and competence. Their role includes mastery of the subject, pre-teaching capabilities, knowledge of learning patterns, fair evaluation, felicity, understanding, ability to transfer the instructional material, assertiveness, seriousness in classroom management, ability to access new information and reason clinically, audit and self-assessment, entrepreneurial and team-building skills, and teamwork, all of which emphasizing the problem-solving and critical thinking skills. Several studies have emphasized the importance of this issue and have considered the duties of teachers and the ability and personality traits to be essential for the role of a professor [4, 30, 31]. The results of the study by Nawabi et al. considered the role of a medical professor beyond the boundaries of information providers; it is emphasized that in modern medical education, the most crucial role of a medical professor is not only an information provider, but also a complete career model and an academic advisor for students [4].

According to the participants in the study, having behavioral traits such as resiliency and good mood, respecting the students, being rational and critical, being humorous, managing the conflicts, being a role model, and having advanced communication skills were necessary for a competent teacher. This means that teachers with good morals and human qualities can contribute to the scientific and moral atmosphere and guide students in science and ethics. According to the experts of the present study, justice is one of the influential factors in empowering a teacher. The human traits and characteristics of teachers have been emphasized in many studies [4, 22, 32].

Algatani et al., in a study at Sultan Qaboos University in Saudi Arabia, identified the needs of teachers and the essential skills that should be considered when planning a program for the development and empowerment of teachers. In addition, they concluded that there were gaps in the current and favorable conditions in medical education in Saudi Arabia. This study also considered the essential component in the effectiveness of a successful teacher development program (FDP) in medical education as an improvement in the personality components of the teacher [33].

The second theme was the best strategies for empowering the teacher with four sub-themes, including problem-based learning, integration of methods, evaluation-based education, and scholarship in education (PIES); accordingly, it can be said that this strategy can be effective in empowerment of the faculty development according to experiences of the academic staff in Iranian universities.

In this relation, Fernandez in a study states that some evaluation models better detect the complexity of the effects of faculty development, in particular the mutual relationships between program components and outcomes [2].

Among the options related to the first category (problem-based education), the methods of familiarity with the latest educational, research, and management findings by raising the problem and teaching consequently are the most important ones. Among the second category options (blended learning), the most important one is the combination of face-to-face and e-learning in terms of content and participants in the training courses. Also, among the third category options (assessment-based education), monitoring and providing feedback on the academic behavior were emphasized, leading to behavior modification in action.

According to the study results, learning is based on the problem-oriented strategies of teachers’ empowerment. Participants considered the real problem to be the expression of the existing problems of the educational environment, which can be involved in the training groups of teachers to engage young and experienced teachers with real problems and try to solve them. Henriksen et al. point out that if participants begin to use the problem and the results of the problem thoughtfully, they will develop new knowledge and skills to improve education and develop problem-solving skills, which can solve many educational problems [34].

It is believed that a problem-based learning environment (PBL) supports a small group, which leads to synergy. In small groups, the development of field-specific knowledge and professional skills, such as teamwork, clinical reasoning and literacy information, continuous assessment, and feedback on these skills can be observed. Roberts has also found that using problem-based techniques in small groups in empowerment workshops is effective [35].

There was integrated training with a combination of participants’ experiences in different forms. The first was a combination of face-to-face and e-learning or a combination of different content and teaching methods. Participants believed that in the affluent world of the 21st century, the obligation to attend programs was futile. Teachers were the people with limited time who could not attend all programs. Numerous studies also indicate that they play an essential role in empowering the teachers in integrated training [36–38]. By using the principles of adult education, blended learning provides greater flexibility and responsiveness in the teaching and learning process[38].

The study results and the experiences of explaining empowerment showed that it was necessary to evaluate the audience and learners to empower the staff. In this study, the emphasis was on formative evaluation, and feedback was presented as a correction. Others emphasized student learning as the end product of teacher development. The educational portfolio was also one of the cases, the impact of which on teacher evaluation was emphasized by the participating experts, showing both dimensions of modifying teacher behavior and ensuring his/her ability [39]. Pyorala at the University of Helsinki in Norway showed how to design a role-based portfolio. This tool is the result of practical research that tries to encourage and support the growth of teachers in various educational roles and be helpful for the professional development of teachers. Participants in the course used reflection, portfolio, feedback, and observational notes to demonstrate their professional competence [40].

Studies show that universities have used different approaches to address this challenge. Holding orientation courses for teachers, especially novice ones, and training courses to update them to provide new teaching materials and teaching aids and simplify the process of implementation of research are among the measures that do not seem to have had much effect. In Shiraz University of Medical Sciences, unique models for educational research such as Eq. 2, including four main components, “teaching, counseling and mentoring, curriculum development, and comprehensive evaluation,“ have been used to promote the faculty members’ abilities [41]. There is also evidence that having personal motivation and a background and experience in education alone are not enough. The support of the institute is necessary to conduct educational research [6].

Some studies point out that medical teachers in different roles must reflect their duties, which will change their practice and professional maturity. This reflection also leads to creativity in teaching and essential thinking skill for creating educational innovation that is valuable and useful in teaching. Many creative educational models have been used in higher education to promote creative thinking [6, 42, 43].

### Implications of the findings

The results of this study can help authorities take measures to empower and assess the faculty members; the strategies found in this study will be useful in designing models for development and empowerment of the faculty members in universities. Moreover, the faculty members’ assessment can be designed based on the findings of this study.

Competent teachers will not use their abilities and skills without satisfaction and respect, the required information and authority, and a positive attitude. Therefore, paying attention to human features and characteristics in empowerment programs is very important from the perspective of the teachers and learners because teachers are a learner in empowerment courses and a teacher in the classroom and educational environment. Blended learning and teacher training programs should be designed to help the lecturers move towards self-assessment, which is also an important issue that puts the responsibility of teachers’ empowerment on themselves as active and mature learners.

Finally, there is a significant shortage of research on health education in the relatively limited literature on faculty empowerment. The scholarship is the key to engaging teachers in educational studies and improving the educational system in the university; it changes the educational settings into an active and dynamic environment.

### Study limitations

Because qualitative studies are subjective, their findings are rooted in data from specific situations or groups. Caution should be exercised in applying these findings to other situations.

## Conclusion

The academic staff have numerous roles and responsibilities, the empowerment of which needs efficient and operational strategies. Based on the participants’ views, their commitment to their responsibilities and meatabilities besides their professional features should be considered by authorities, so that they can be a competent and efficient lecturer. To the best of our knowledge, no study has been conducted on strategies used for the faculty members’ empowerment based on their roles and duties; therefore, the results of the present study can help all those involved in teahing in universities.

Based on the results, the best strategies for empowering the teacher with four sub-themes included problem-based learning, integration of methods, evaluation-based education, and scholarship in education (PIES); accordingly, it can be said that this strategy can be effective in empowerment of the faculty members in Iranian universities. Moreover, it was found that if we engage the teachers in solving educational and research problems in the educational environment in an integrated manner as it happens in the real world, learning and motivation are promoted and evaluated in the academic environment. We recommend that empowerment and ssessment of the faculty members should be further studied in future research.

## Electronic supplementary material

Below is the link to the electronic supplementary material.

Supplementary Material 1

## Data Availability

All the data of this study are available upon request from the corresponding author.

## List of abbreviations

SUMS: Shiraz University of Medical Sciences
EDC: Educational Development Cente
EMRO: Eastern Mediterranean Regional Office
PBL: problem based learning
