# Supplementary material for: Faculty development strategies to empower university teachers by their educational role: A qualitative study on the faculty members and students’ experiences at Iranian universities of medical sciences
Source: BMC Med Educ. 2023 Apr 19;23:260. doi: 10.1186/s12909-023-04209-0 (PMC10114459; doi:10.1186/s12909-023-04209-0)
Supplement: Supplementary file 1 — Supplementary Material 1 [file 12909_2023_4209_MOESM1_ESM.docx]

A guide to interview questions for medical science teachers' empowerment strategies in relation to their experiences regarding the duties of a university professor

Demographic questions: age, education and academic rank, and work experience in which faculty and educational group they work

Question: What are the roles of a university professor in the university?

Question: What are the educational roles of a university professor in the university?

Question: What are your educational roles as a university professor?

Question: Explain your experience or an example of your roles.

Question: As a university professor, what are your duties in your educational role?

Question: As a university professor, what kind of training do you need to fulfill your educational role, for example, as a lecturer or evaluator in student evaluation?

Question: What topics, roles, duties, or tasks have you been trained in so far?

Question: What points did the teachers emphasize?

Question: What was the teaching method of the teachers?

Question: In your opinion, what points should be followed in empowering teachers to get better results?

Question: Did evaluations also take place in these teacher empowerment courses? How?

Question: Has a pre-training needs assessment been done regarding your educational needs?

Question: In general, how do you evaluate the training programs for empowering professors in their teaching roles?

Question: What are your suggestions for improving the training programs for the empowerment of teachers; please give an example.
